# Gastroparesis in patients with inactive Crohn's disease: a case series

**DOI:** 10.1186/1471-230X-7-11

**Published:** 2007-03-21

**Authors:** Jón O Kristinsson, Wim PM Hopman, Wim JG Oyen, Joost PH Drenth

**Affiliations:** 1 Department of Gastroenterology and Hepatology, Radboud University Medical Center, Nijmegen, The Netherlands; 2 Department of Nuclear Medicine, Radboud University Medical Center, Nijmegen, The Netherlands

## Abstract

**Background:**

Few studies have described patients with foregut dysmotility in inflammatory bowel disease. The aim of this case series was to evaluate clinical characteristics of 5 patients with inflammatory bowel disease and symptoms and signs of upper gut dysmotility.

**Case presentations:**

We describe a series of four patients with Crohn's disease and one with indeterminate colitis who presented with severe symptoms and signs of gastroparesis. We reviewed medical records of all cases. Gastric emptying of a solid meal was assessed by scintigraphy. Small bowel enteroclysis, gastroduodenoscopy and colonoscopy with biopsies were performed to estimate the activity of the disease and to exclude organic obstruction. None of the patients had any signs of active inflammation or stricture. All of the patients had markedly delayed gastric emptying with a mean t 1/2 of 234 minutes (range 110–380 minutes; normal values 54–94 minutes).

**Conclusion:**

Clinicians should consider impaired gastric emptying when evaluating patients with Crohn's disease and severe symptoms of upper gut dysmotility, which cannot be attributed to active inflammation or organic obstruction of the digestive tract. Symptoms in these patients are refractory to various therapeutic interventions including tube feeding and gastric surgery.

## Background

Crohn's disease is a chronic inflammatory condition of unknown aetiology, which may involve the whole digestive tract from the oral cavity to the anus. Symptoms indicative of gastroparesis, such as vomiting and bloating do occur in patients with Crohn's disease, but recurrent and/or persistent symptoms are rare and most clinicians attribute them to active inflammation or mechanical obstruction of the small or large bowel. Though local inflammation and intestinal obstruction may have important effects on gastrointestinal motility, motility studies in patients with Crohn's disease are scant [1,2]. Some reports indicate that upper gut motility can be impaired in patients with inactive Crohn's disease [1,3].

The aim of this case series is to describe clinical characteristics of five patients (four patients with inactive Crohn's disease and a single patient with indeterminate colitis) who had severe symptoms of gastroparesis which could not be attributed to mechanical obstruction or active inflammation, but who all had impaired gastric emptying of solid food.

## Case presentation

The patients were all treated in our institute which is a tertiary referral centre for inflammatory bowel disease (IBD) in the Netherlands. We identified patients by a search of the morbidity database of the department of Gastroenterology and Hepatology which includes the ICD-9 diagnoses of975 patients with Crohn's disease and 821 patients with colitis ulcerosa referred between 1973 and 2004 and by a search of the gastric emptying database which includes 239 patients who underwent a scintigraphic gastric emptying study in our hospital between 1999 and 2004. We selected cases with a diagnosis of IBD including Crohn's disease and ulcerative colitis who also had an impaired gastric emptying by matching the two data bases. We identified four patients with Crohn's disease and one with indeterminate colitis who were diagnosed based upon accepted radiological, endoscopic and histological criteria [4]. We have reviewed the medical records of the cases who all had a severe symptoms of foregut dysmotility.

Because of persistent symptoms of nausea, vomiting and in some cases weight loss patients were thoroughly evaluated. All patients underwent a small bowel enteroclysis. None of the patients had signs of active inflammation or stricture. Colonoscopy with inspection of the terminal ileum was performed in every patient and biopsy specimens were taken for pathological examination. All patients underwent an upper gastrointestinal endoscopy to rule out active Crohn's disease in the upper digestive tract and intestinal stenosis as cause of the symptoms. Random biopsies were taken from the gastric antrum and corpus. We found no mechanical obstruction in any of the patients and there were no endoscopical or histological signs of active Crohn's disease. Furthermore there were no signs of inflammation in the laboratory studies. After having ruled out active inflammatory disease gastric emptying was evaluated by a scintigraphy in all patients.

Table 1 summarizes clinical data of all patients. Remarkably all of them were females with a mean age of 38.6 years at the time of impaired gastric emptying (range 21 – 56 years). The mean duration of the disease was 9.4 years with a wide range from 2 to 26 years. One patient had small bowel involvement, in two the disease involved the small bowel and the colon, and in two it was limited to the colon. Two of the patients had undergone resection, colectomy with ileostomy (patient 4) and ileum resection twice (patient 2). Despite discouragement, three of the patients were smoking. The mean t 1/2 of gastric emptying was 234 minutes (range 110–380 minutes).

We describe one of the cases (case1) in more detail. This was a 19-yr-old female patient with a history of asthma presented to our outpatient clinic with complaints of abdominal pain and chronic diarrhea that had been present for approximately 5 months. She complained of continuous pain localized in the upper abdomen but she also had intermittent colic-like pain elicited by food ingestion, sometimes accompanied by nausea. There was watery diarrhea with a frequency of up to 10 times a day. She had anorexia and lost 15 kg of weight in the preceding 5 months. She had been taking diclofenac and she smoked 4 cigarettes per day.

On physical examination she had normal vital signs. Her weight was 84 kg with a length of 171 cm. There was a mild tenderness in the right lower abdomen. Further examination was not remarkable.

Initial laboratory studies revealed a sedimentation rate of 25 mm in the 1st hr, C-reactive protein of 40 mg/L, albumin 37 g/L (normal 36–53 g/L), haemoglobin was 7.8 mmol/L (normal 7.3–9.7 mmol/L), white cell count was 12.1 × 10^9^/L (normal 3.5–11.0 × 10^9^/L) with 75% neutrophils and thrombocytes were elevated (480 × 10^9^/L; normal 120–350 × 10^9^/L). Sigmoidoscopy revealed a patchy erythema with rectal sparing. Histopathology of the biopsy specimen showed chronic active inflammation without presence of granulomas. Barium examination of the small bowel demonstrated a narrowing of the distal ileum with thickening of the wall over a length of 40 cm.

A diagnosis of Crohn's disease with involvement of left colon and terminal ileum was made and treatment was started with oral corticosteroids and azathioprine in combination with mesalazine. Because of adverse reaction (fever) to azathioprine and thereafter mercaptopurine, tioguanine was prescribed, and well tolerated.

One year and a half after the first presentation she complained of nausea, vomiting, early satiety, weight loss of 15 kg and diarrhea. Laboratory studies now showed no signs of inflammation. There were no endoscopic signs of activity of Crohn's disease in colon, terminal ileum or stomach and duodenum. Biopsy specimens taken from the gastric antrum, duodenum, terminal ileum and colon revealed no active inflammation. Small bowel enteroclysis showed no stricture and the terminal ileum was now normal. Gastric emptying with radiolabeled pancake was severely delayed with a t 1/2 of 200 minutes and 3 months later with a t 1/2 of 380 minutes.

Tioguanine was stopped because of the remote possibility that the symptoms were secondary to the drugs, but this did not ameliorate the symptoms. Because of persistent foregut dysmotility symptoms nasojejunal feeding was initiated. Recently she underwent a Roux-Y gastrojejunostomy with introduction of an enterocutaneous jejunal feeding catheter.

## Treatment and prognosis

Patient 1 is the only patient who underwent surgery because of her foregut dysmotility. The surgical procedure was recently performed but at present the gastroenterostomy fails to improve her symptoms. Patient 4 was only treated with oral prokinetics that had a partial effect while all other patients also received nasogastric 24 hour continuous drip tube feeding. One of these three patients (patient 3) underwent a percutaneous endoscopic gastrostomy tube (PEG) placement, which was removed two months later for psychological reasons. Despite these interventions, symptoms of upper gut dysmotility persist in all patients.

## Discussion

This report describes five patients with clinically inactive inflammatory bowel disease who presented with persistent severe complaints of upper gastrointestinal motility disorder. All patients had disabling upper gastrointestinal symptoms with weight loss which in most of the cases led to invasive treatment including nasogastric tube feeding, PEG and even gastrojejunostomy with placement of a jejunostomy catheter.

Reports on delayed gastric emptying in Crohn's disease are scarce. Annese et al studied gastric emptying in 21 adult patients with nonobstructive Crohn's disease [2]. Gastric emptying was not different from that in healthy volunteers. Only posthoc analysis revealed impaired gastric emptying in a subgroup of patients who complained of mild upper gut symptoms such as bloating, early satiety and abdominal distention and in those with localization restricted to the colon [2]. Our case series extends these findings and shows that delayed gastric emptying in inactive Crohn's disease may induce serious upper gut symptoms prompting clinicians for elaborate diagnostic investigations including upper gut endoscopy and assessment of gastric emptying. Our series shows that delayed gastric emptying is not confined to patients with localization restricted to the colon.

Why do patients with Crohn's disease develop symptoms of foregut dysmotility? It is logical to infer that active Crohn's disease with accompanying intestinal inflammation has functional ramifications. Contraction of smooth muscle from inflamed small intestine derived from patients with Crohn's disease is abnormal [3]. Distal motility disturbances due to ileal or colonic inflammation might subsequently impair gastric emptying, a phenomenon similar to that in patients with slow transit constipation [5,6]. Likewise, obstruction of the small bowel or colon due to Crohn's disease can cause foregut dysmotility. Moreover, gastric Crohn's disease impairs gastric emptying which can be observed in up to 70% of cases, but this is more often seen in children as compared to adults [7-9]. None of patients described in this report had signs of active disease. Enteroclysis, colonoscopy, endoscopy of the upper gut with bioptic samples and laboratory studies were compatible with absence of active inflammation in all patients.

Two of our patients had a history of intestinal resection. However, surgical resections are not associated with a detrimental effect on gastric emptying in patients with Crohn's disease [10].

Antroduodenal manometry studies have also shown that upper gastrointestinal motor disorders occur in up to 74% of patients with uncomplicated Crohn's disease [1]. We did not perform an antroduodenal manometry in any of the patients in this case series.

The explanation for the foregut dysmotility and the related symptoms of our patients is not clear. It is possible that there is a minor degree of intestinal fibrosis without radiological signs of obstruction or inflammation of the bowel that cannot be detected with standard clinical methods. On the other hand, the gastrointestinal motility is regulated by gut hormones, of which peptide YY (PYY) is the most prominent member [11]. Circulating PYY inhibits gastric emptying [12,13]. It has been shown that patients with Crohn's disease have elevated serum levels of PYY [14], which could be a possible explanation for upper gastrointestinal motility disturbance in these patients. Lastly, Porcher et al [15] reported that interstitial cells of Cajal were abundant in the small intestine of patients with Crohn's disease and reasoned that this could lead to desynchronization of electrical pacemaker activity. This might be due to invasion of the external muscle layers by inflammatory cells.

## Conclusion

We describe five patients with Crohn's disease who had no signs of active inflammation but developed severe symptoms of gastroparesis. It remains to be elucidated why some patients with inactive Crohn's disease develop upper gut motor disturbances. According to our experience these patient will develop persistent symptoms despite medical treatment. Tube feeding is most often necessary and even bypassing of the stomach seems to be the only therapeutic possibility in the most extreme cases.

## Competing interests

The author(s) declare that they have no competing interests.

## Authors' contributions

JK: Drafting, conception and design of the manuscript.

WH: Critical revision of the manuscript, assistance in collection of data.

WO: Critical revision of the manuscript, assistance in collection of data.

JD: Conception, design and drafting of the manuscript

All authors read and approved the final manuscript.

## Pre-publication history

The pre-publication history for this paper can be accessed here:



## Figures and Tables

**Table 1:** Clinical data of the five patients

Patients	1	2	3	4	5
Diagnosis	CD	CD	CD	CD	IC
Sex	F	F	F	F	F
Age (yr)	21	29	38	56	57
Age at diagnosis (yr)	19	23	28	30	54
BMI (kg/m2)	37,4	33	31,4	22,3	19
Localisation	Ileum-Colon	Ileum	Ileum-Colon	Colon	Colon
Resection	No	Ileum × 2	No	Colectomy	No
Stomy	No	No	No	Ileostomy	No
Smoking	Yes	Yes	Yes	No	No
T 1/2 (min)	380	288	220	110	171

CD, Crohn's disease; IC, indeterminate colitis; BMI, body mass index
